# Comprehensive visual electrophysiological measurements discover crucial changes caused by alcohol addiction in humans: Clinical values in early prevention of alcoholic vision decline

**DOI:** 10.3389/fncir.2022.912883

**Published:** 2022-08-11

**Authors:** Xin Xie, Kang Feng, Juan Wang, Min Zhang, Jing Hong, Haolin Zhang

**Affiliations:** ^1^Beijing Key Laboratory of Restoration of Damaged Ocular Nerve, Peking University Eye Center, Peking University Third Hospital, Beijing, China; ^2^Faculty of Environment and Life, Beijing University of Technology, Beijing, China

**Keywords:** alcohol addiction, electrophysiology of vision, electroretinogram (ERG), visual evoked potential (VEP), multivariate statistical analyses (MVA)

## Abstract

Alcohol addiction often compromises vision by impairing the visual pathway, particularly the retina and optic nerve. Vision decline in alcoholics consists of a sequential transition from reversible functional deterioration of the visual pathway to irreversible clinical vision degeneration or vision loss. Thus, the control of alcoholic vision decline should focus on prevention before permanent damage occurs. Visual electrophysiology is a promising method for early detection of retinal dysfunction and optic neuropathy, including full-field electroretinography (ffERG) and pattern-reversal visual evoked potential (PR-VEP). So far, however, research studying the electrophysiological characteristics in the preclinical stage of vision decline caused by alcohol addiction is still lacking. Here we conducted a retrospective study with 11 alcoholics and 14 matched control individuals to address this need. We had performed comprehensive visual electrophysiological tests, including ffERG and PR-VEP. We next analyzed all electrophysiological parameters using multivariate statistical analyses and discovered some highly sensitive alterations to alcohol addiction. We found severely reduced amplitudes in scotopic ffERG oscillatory potentials (OPs) in alcohol addicts. These changes indicate the alcohol-induced disturbances of amacrine cells and retinal circulation. In subjects with alcohol addiction, the amplitudes of b-waves diminish significantly in scotopic but not photopic ffERG, implying the impaired function of the retinal rod system and the dysfunction of the inner retina. PR-VEPs elicited by checkerboard stimuli with large 1 degree (°) checks mainly reflect the state of the optic nerve and ganglion cells, and PR-VEPs provoked by small 0.25° checks mainly reflect the function of the macular. We performed both measurements and observed a robust amplitude reduction in all three peaks (N75–P100, P100–N135) and a significant peak time extension in P100. Our research provides an affordable and non-invasive tool to accurately evaluate visual pathway conditions in alcohol addicts and help clinicians take targeted treatment.

## Introduction

Alcohol addiction induces pathological alterations not only in reward-associated neural circuits (Gilpin and Koob, 2008; Vengeliene et al., 2008) but also in neural circuits that control visual function (Karimi et al., 2021). The disturbances in alcohol-induced visual pathway dysfunction can lead to retinopathy and optic neuropathy, resulting in vision decline (Brasil et al., 2015; Creupelandt et al., 2021). Although permanent vision impairment induced by intemperate consumption of alcohol is preventable, alcoholics do not usually perceive the functional damage to their eyes until clinical symptoms or organic diseases appear. Some studies indicate that chronic alcohol intake first induces functional disorders reflected in the decreased sensitivity of the visual cells and reduced conductivity of the optic nerve without causing anatomical changes within the retina and the optic nerve (Ozsoy and Alim, 2020; Karimi et al., 2021). Therefore, establishing an effective method that can detect the functional deterioration of the visual pathway early is highly necessary for controlling alcoholic vision decline.

The eye is an essential neurosensorial organ that is more easily accessible and detectable than the brain. Recording the functional alterations of the visual pathway caused by alcohol addiction by non-invasive visual electrophysiology can directly reflect the impairments of neural circuits and effectively monitor the progression of alcohol-induced deficits in visual function. The full-field electroretinogram (ffERG) is a graphic tracing of responses from the retina upon stimulation by light and is of value in studying retina diseases and can be used as an objective measurement of retinal function. It records changes in different modes to reflect the function of various retinal cell types (McCulloch et al., 2015). Visual evoked potentials (VEPs) are visually evoked electrophysiological signals extracted from the electroencephalographic activity in the visual cortex recorded from the overlying scalp. VEPs depend on the functional integrity of central vision at all levels of the visual pathway, including the eye, retina, optic nerve, optic radiations, and the occipital cortex (Odom et al., 2016). Therefore, using these methods to quantitatively monitor critical parameters prone to change in alcohol use has potential clinical application value.

To date, research on the effects of alcohol use on the visual pathway is very limited (Knave et al., 1974; Kim et al., 2016), and many unknown areas need further investigation. First, the existing studies only utilized a small portion of available visual electrophysiological measurements, but discovering crucial changes caused by alcohol addiction requires more comprehensive visual electrophysiological measurements and data analyses. Second, because visual electrophysiological parameters are usually large in amount and are not normally distributed (e.g., ffERG b-wave peak time) (McCulloch et al., 2015; Odom et al., 2016), establishing a more accurate multivariate non-parametric statistical method is necessary to interpret such bulk visual electrophysiological data comprehensively.

To address these questions, here in this study, we focus on human alcohol addiction and assess their visual pathway dysfunction by adopting comprehensive visual electrophysiological methods. We also unselectively incorporated all acquired electrophysiological parameters and performed an unbiased, detailed and objective evaluation and analysis of the visual pathway of alcohol addiction patients. The data were analyzed using multivariate statistical methods and presented in visual forms. Our research aims to optimize the management of chronic alcohol consumption and its consequences, such as optic neuropathy, to preserve a higher level of vision and visual function.

## Materials and methods

### Study subjects and ethics

In this retrospective study, all the cases were selected from out-patient individuals who had undertaken one of the ffERG and pattern-reversal visual evoked potential (PR-VEP) or both between September 14, 2017, and March 24, 2022, at the Department of Ophthalmology of Peking University Third Hospital. Alcohol addiction individuals were selected based on the following inclusion criteria: (1) A history of chronic wine alcoholism for at least 2 years, drinking alcohol (alcohol content of 40–50%) at least three times a week, and at least 100 ml per drink. (2) No non-alcoholic vitreoretinal diseases, no non-alcoholic optic nerve diseases, and no other non-alcoholic ocular diseases. The inclusion criteria for the control group are no vitreoretinal diseases, no optic nerve diseases, and no other ocular diseases. Cases were excluded if they had uncorrectable ophthalmic comorbidities which can influence their visual response to stimuli in electrophysiological examinations. We controlled for smoking status, age, and gender to make the two groups comparable. The study was approved by the institutional review board (approval number: 119-03). All procedures performed in this study were in accordance with the Peking University Third Hospital ethics committee and with the 1964 Helsinki declaration and its later amendments.

### ffERG test

**FIGURE 1:**
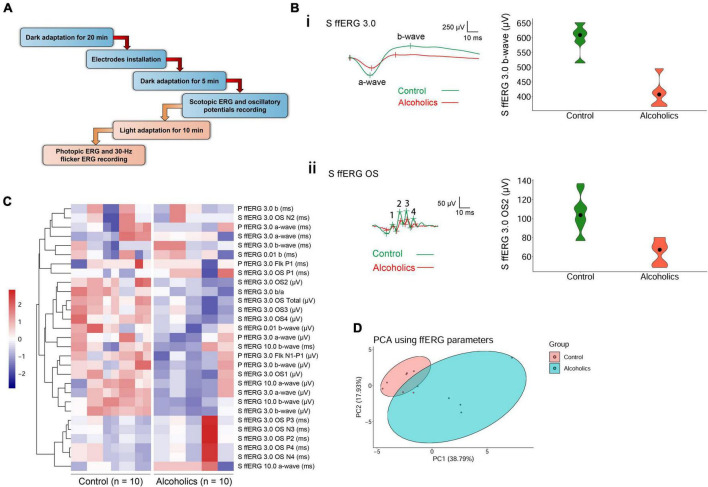
ffERG shows significant differences between alcoholics and control individuals. **(A)** Experimental procedures are summarized in the diagram. **(B)** Typical ffERG waveforms in normal and alcohol addictive individuals are presented, and quantification of representative parameters are displayed in the form of violin plots. (i) Scotopic ERG 3.0 waveforms and the quantification and distribution of b-wave amplitudes are shown. (ii) Scotopic ERG 3.0 oscillatory potential wavelets and the quantification and distribution of OS2 amplitudes are shown. In scotopic ERG oscillatory potentials, the first peak was identified as OP1. OPs 2–4 following OP1 were labeled in sequential order. The inserts give scale. **(C)** Heatmap reveals many reduced amplitudes in alcohol addicts by ffERG measurements. Correlated parameters are clustered. **(D)** PCA combining data from ffERG parameters shows variation between the normal and alcoholics across PC1 and PC2. S, scotopic; P, photopic; OS: oscillatory; Flk: flicker.

Electroretinograms were measured according to the standard International Society for Clinical Electrophysiology of Vision (ISCEV) protocol (McCulloch et al., 2015). Before the test, participants received pupil dilation for at least 20 min until their pupil diameter reached 8 mm, followed by a dark adaptation for another 20 min. Then, scotopic ERG and dark-adapted oscillatory potentials (OPs) were recorded. After the dark-adapted ERGs, the eyes were light-adapted for 10 min, and the photopic ERG and 30-Hz flicker ERG were recorded. Both eyes were recorded simultaneously. A Ganzfeld stimulator (RETI-port/scan 21, Roland, Berlin) delivered flash stimulus. Contact lens electrodes recorded ERG. Electrodes were lying across the center of the cornea of each eye moistened with 1% carboxymethylcellulose sodium. Reference and ground gold disc electrodes were kept with adhesive paste at the external canthi and forehead. We used the Ganzfeld electrophysiology system for stimulus generation, data acquisition, and data processing. A brief illustration of the above procedures is shown in Figure 1A.

### Pattern-reversal visual evoked potential test

Pattern-reversal visual evoked potentials were measured according to the ISCEV protocol (Odom et al., 2016). VEPs result from stimulation of each eye individually. Checkerboard pattern reversal stimuli elicited PR-VEPs with large 1 degree (°) and small 0.25° (15′) checks. PR-VEPs used a reversal rate of 2.0 ± 0.2 reversals per sec (rps), and this corresponds to 1.0 ± 0.1 Hz, as a full cycle includes two reversals. All the patients examined had visual acuity of >0.1.

### Digital color photographs of the fundus

Digital color photographs of the fundus (ETDRS standard field 1, centered on the disc) were taken using a non-mydriatic retinal camera (CR-2 AF; Canon, Japan) based on the standardized protocols.

### Statistical analyses

We used data from both eyes unless one eye had other clinical abnormalities such as injury. In cases when subjects have single-eye data, we duplicated the values of the available eye so that all subjects have a pair of results. This strategy has considered the correlation between eyes from the same subjects, can achieve more accurate estimations, and is proved better than using only one eye’s data by our study and also by literature (Davis and Hamilton, 2021). All statistical analysis and data visualization were performed using R software. Data were first assessed for normality using the MVN package (Korkmaz et al., 2014). The vegan package was used to perform Permutational Multivariate Analysis of Variance (PERMANOVA) to compare overall changes in ffERG and PR-VEP between the two groups. Shapiro–Wilk test was used to test univariate normality of individual parameters. Unpaired student’s *t*-test was used for testing differences in individual electrophysiological parameters that followed univariate normal distribution and with homoscedasticity. Wilcoxon rank sum test was utilized for testing differences in individual electrophysiological parameters that followed univariate non-normal distribution. Bonferroni correction was used to correct for false positive. Violin plots and PCA plots were constructed using the ggplot2 package. Heatmaps were constructed using the pheatmap package.

## Results

### Multivariate statistical analyses reveal a significant difference in ffERG and pattern-reversal visual evoked potential properties between alcoholics and control individuals

**FIGURE 2:**
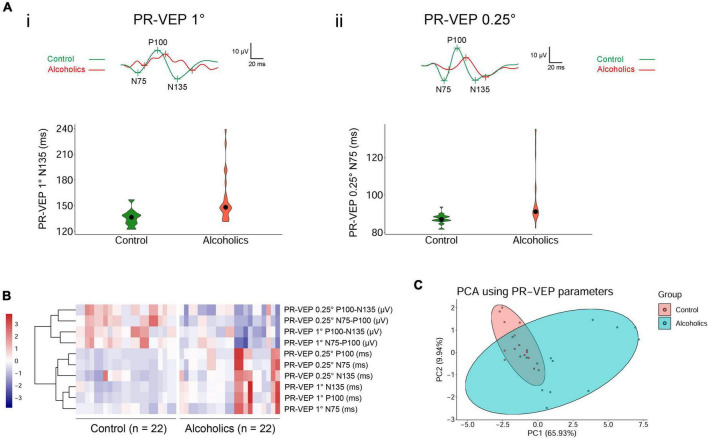
Pattern-reversal visual evoked potential (PR-VEP) shows significant differences between control individuals and alcoholics. **(A)** Typical PR-VEP waveforms in normal and alcohol addictive individuals are presented, and quantification of representative parameters are displayed in the form of violin plots. (i) PR-VEP 1° waveforms and the quantification and distribution of N135 peak times are shown. (ii) PR-VEP 0.25° waveforms and the quantification and distribution of N75 peak times are shown. **(B)** Heatmap reveals that all amplitudes are reduced and most peak times are prolonged in alcohol addicts by PR-VEP measurements. Correlated parameters are clustered. **(C)** PCA combining data from PR-VEP parameters shows variation between the normal and alcoholics across PC1 and PC2.

We had performed comprehensive visual electrophysiological tests, including ffERG and PR-VEP in 11 patients with alcohol addiction and 14 age- and gender-matched normal control individuals. The individuals with alcohol addiction had drinking histories ranging from 2 to 40 years, with the median of 20 years. According to the Diagnostic and Statistical Manual of Mental Disorders, Fifth Edition (DSM-5) and International Classification of Diseases, Tenth Revision (ICD-10), all the alcohol addicts in this study were diagnosed as moderate to severe alcohol use disorder (AUD). A summary of the demographic and clinical data of the participants is presented in Table 1. We acquired 29 electrophysiological parameters for ffERG and 10 electrophysiological parameters for PR-VEP. The statistical normality test revealed that all the data follow the multivariate non-normal distribution (ffERG: *H* = 83.88, *p* = 1.49 × 10^–12^; PR-VEP: *H* = 84.53, *p* = 1.59 × 10^–16^; Royston test), like the visual electrophysiological data described previously (McCulloch et al., 2015; Odom et al., 2016). Univariate normality tests and tests of homogeneity of variance for individual parameters revealed that around half of the parameters followed non-normal distribution (Supplementary Tables 1a,b) and around half of the parameters did not meet homoscedasticity (Supplementary Tables 2a,b). PERMANOVA revealed a significant difference between patients with alcohol addiction and control individuals (ffERG: *F* = 22.40, *p* = 2.00 × 10^–4^; PR-VEP: *F* = 14.58, *p* = 1.00 × 10^–4^; PERMANOVA). Heatmap (Figures 1C, 2B) and PCA (Figures 1D, 2C) also presented the differences visually. In the heatmap, correlated parameters were clustered, and the clustering by statistics is in accordance with the similarity of parameters by their electrophysiological characteristics. For example, in PR-VEP, parameters of amplitudes and parameters of peak times were clustered, respectively (Figure 2B).

**TABLE 1:** Demographics and clinical data of the study subjects.

Characteristic	Control, *n* = 28	Alcoholics, *n* = 22	*P*-value
Test date	2018-05-16 to 2022-01-24	2017-09-14 to 2022-03-24	
Gender			
Male	14 (100%)	11 (100%)	
Age	44 (34, 48)	49 (34, 54)	0.10
Smoking			0.9
No	10 (36%)	8 (36%)	
Yes	6 (21%)	6 (27%)	
Unspecified	12 (43%)	8 (36%)	

Data are expressed as the median (P25, P75). n refers to the number of eyes examined.

Differences in age were tested using Wilcoxon rank sum test; differences in smoking status were tested using Pearson’s Chi-squared test.

### Scotopic ffERG wave amplitudes are highly sensitive to chronic alcohol consumption

A novel finding by screening the ERG data is that there were statistically significant differences between the alcoholic and normal control groups in scotopic ERG OPs, which represent responses primarily from amacrine cells. Specifically, most of the oscillation potential components (OP2–OP4) were significantly reduced in amplitude, but no peaks of OP1–OP4 increased in peak time in the alcohol addiction group (Figure 1Bii and Table 2). We measured the difference in rod activity by scotopic ERG 0.01 responses. The b-wave amplitude displayed a trend of reduction in alcoholic subjects compared to those of the control subjects, but was not significantly different (Table 2). Dark-adapted ERG 3.0 and 10.0 b-wave responses arise from bipolar cells, and both 3.0 and 10.0 b-wave amplitudes, together with b-wave/a-wave ratio, were significantly lower in subjects of alcohol addiction than that in control individuals (Figure 1Bi and Table 2). No significant difference in 3.0 or 10.0 b-wave peak time was observed between subjects of alcohol addiction and control individuals (Table 2). We assessed the difference in cone function by photopic ffERG. Light-adapted 3.0 a- and b-wave amplitude in alcoholics compared to healthy controls showed a trend of decrease, but had no significant difference (Figure 1C and Table 2). No change was observed in light-adapted 3.0 a- and b-wave peak times (Table 2). By photopic flicker ERG, ERG amplitudes (N1-P1, μV) showed a downtrend with no statistical significance (Figure 1C and Table 2). Peak time values were comparable between subjects of alcohol addiction and healthy control individuals (Table 2). Overall, alcohol addiction proved to have a greater effect on rod system than the cone system, and a greater effect on inner retina than the photoreceptors. Furthermore, there was significant difference between alcohol addiction and the control group for many of the amplitudes of ERG parameters but for none of the peak times, indicating that ERG changes feature amplitude reduction but not peak time extension.

**TABLE 2:** ffERG components statistics.

Characteristic	Control, *n* = 10	Alcoholics, *n* = 10	*P*-value
S ffERG 0.01 b-wave (ms)	83.01 ± 5.41	83.46 ± 4.88	0.85
S ffERG 0.01 b-wave (μV)	307.50 ± 62.49	229.80 ± 46.24	5.41 × 10^–3^
S ffERG 3.0 a-wave (ms)	18.35 (17.75, 22.00)	19.70 (19.40, 19.70)	0.88
S ffERG 3.0 b-wave (ms)	46.40 (46.10, 46.70)	47.30 (45.80, 55.50)	0.29
S ffERG 3.0 a-wave (μV)	349.00 (332.00, 354.00)	248.00 (246.00, 280.00)	0.02
S ffERG 3.0 b-wave (μV)	609.50 (587.00, 625.00)	407.00 (373.00, 419.00)**	1.50 × 10^–4^
S ffERG 3.0 b/a	1.80 ± 0.15	1.52 ± 0.09**	9.27 × 10^–5^
S ffERG 10.0 a-wave (ms)	16.25 (15.90, 16.63)	17.30 (17.30, 17.30)	0.02
S ffERG 10.0 b-wave (ms)	49.50 (48.10, 51.00)	40.20 (39.00, 49.30)	0.36
S ffERG 10.0 a-wave (μV)	389.00 (376.00, 435.00)	311.00 (286.00, 333.00)	0.05
S ffERG 10.0 b-wave (μV)	617.90 ± 55.69	429.00 ± 49.54****	2.39 × 10^–7^
S ffERG 3.0 OS P1 (ms)	18.50 (18.28, 18.50)	18.80 (18.50, 18.80)	0.07
S ffERG 3.0 OS N2 (ms)	21.40 (21.40, 21.70)	21.40 (21.40, 21.40)	0.75
S ffERG 3.0 OS P2 (ms)	24.40 (24.40, 25.00)	25.20 (24.70, 25.50)	0.02
S ffERG 3.0 OS N3 (ms)	28.20 (27.60, 28.80)	28.80 (28.20, 29.10)	0.13
S ffERG 3.0 OS P3 (ms)	31.40 (30.20, 31.40)	31.10 (31.10, 32.00)	0.54
S ffERG 3.0 OS N4 (ms)	34.30 (34.10, 36.10)	34.90 (34.60, 35.50)	0.45
S ffERG 3.0 OS P4 (ms)	37.60 (37.08, 39.60)	37.90 (37.60, 39.30)	0.54
S ffERG 3.0 OS1 (μV)	40.00 (39.00, 44.00)	22.00 (21.00, 29.00)	3.96 × 10^–3^
S ffERG 3.0 OS2 (μV)	104.55 ± 20.23	64.36 ± 12.41**	4.34 × 10^–5^
S ffERG 3.0 OS3 (μV)	72.38 ± 14.59	37.46 ± 18.27**	1.70 × 10^–4^
S ffERG 3.0 OS4 (μV)	39.52 ± 9.22	20.38 ± 6.75**	4.92 × 10^–5^
S ffERG 3.0 OS Total (μV)	257.70 ± 35.23	148.78 ± 38.67***	3.48 × 10^–6^
P ffERG 3.0 a-wave (ms)	14.70 (14.40, 15.53)	14.40 (14.40, 14.40)	0.24
P ffERG 3.0 b-wave (ms)	29.90 (29.60, 30.80)	29.60 (29.40, 30.20)	0.76
P ffERG 3.0 a-wave (μV)	73.84 ± 14.46	53.92 ± 16.64	0.01
P ffERG 3.0 b-wave (μV)	200.00 (194.00, 207.00)	112.00 (86.00, 125.00)	0.02
P ffERG 3.0 Flk P1 (ms)	61.10 (59.90, 64.03)	61.60 (59.60, 61.60)	0.82
P ffERG 3.0 Flk N1-P1 (μV)	158.20 ± 19.23	124.08 ± 27.54	4.83 × 10^–3^

Normally distributed data are expressed as the mean ± s.e.m. Non-normally distributed data are expressed as the median (P25, P75).

Significant differences with Bonferroni correction of alcohol addiction group from the control group are indicated (**p < 3.33 × 10^–4^, ***p < 3.33 × 10^–5^, ****p < 3.33 × 10^–6^, unpaired student’s t-test or Wilcoxon rank sum test). S, scotopic; P, photopic; OS: oscillatory; Flk: flicker.

### Pattern-reversal visual evoked potentials display a robust amplitude reduction and a significant peak time extension in alcohol addiction individuals

The alcohol addicts showed remarkable abnormalities in amplitude and peak time of 1° and 0.25° spatial frequency of PR-VEP waves. Specifically, in 1° PR-VEP, P100 and N135 peak times were prolonged, and N75-P100, P100-N135 amplitudes were significantly decreased (Figures 2Ai,B and Table 3), reflecting optic nerve and ganglion cell dysfunction. The decrease in N75-P100, P100-N135 amplitudes was also seen in 0.25° PR-VEP, and the decrease in N75-P100 amplitude was even more significant (Figures 2Aii,B and Table 3). In addition, in 0.25° PR-VEP, P100 peak time extension became much more remarkable (Figures 2Aii,B and Table 3). Alcohol addiction showed a differential effect on 1° and 0.25° PR-VEP N75 and N135 peak time. Specifically, in 1° PR-VEP, N75 peak time did not change but N135 peak time was prolonged (Figures 2Ai,B and Table 3). But in contrast, in 0.25° PR-VEP, N135 peak time did not change but N75 peak time was prolonged (Figures 2Aii,B and Table 3). 0.25° PR-VEP proved to be more sensitive than 1° PR-VEP, because a higher degree of statistical significance was reached for some parameters (Table 3). These findings support a robust effect of alcohol consumption on optic nerve transduction and ganglion cell excitability.

**TABLE 3:** Pattern-reversal visual evoked potential (PR-VEP) components statistics.

Characteristic	Control, *n* = 22	Alcoholics, *n* = 22	*P*-value
PR-VEP 1° N75 (ms)	76.30 (72.03, 78.85)	78.08 (69.90, 89.45)	0.23
PR-VEP 1° P100 (ms)	104.50 (102.85, 106.23)	113.30 (108.83, 123.51)**	1.87 × 10^–4^
PR-VEP 1° N135 (ms)	136.25 (129.03, 139.10)	147.90 (139.90, 160.00)**	3.25 × 10^–4^
PR-VEP 1° N75-P100 (μV)	11.30 (10.13, 12.00)	8.43 (6.74, 9.64)**	1.07 × 10^–4^
PR-VEP 1° P100-N135 (μV)	13.50 (12.00, 17.53)	9.92 (6.16, 11.05)**	2.72 × 10^–4^
PR-VEP 0.25° N75 (ms)	86.90 (86.30, 88.48)	91.00 (89.80, 101.40)****	6.24 × 10^–6^
PR-VEP 0.25° P100 (ms)	107.40 (106.95, 110.40)	116.50 (111.50, 141.00)***	9.01 × 10^–5^
PR-VEP 0.25° N135 (ms)	143.20 (138.73, 153.90)	156.03 (150.30, 183.20)	0.01
PR-VEP 0.25° N75-P100 (μV)	18.08 (11.75, 19.00)	8.09 (5.27, 9.40)****	9.18 × 10^–7^
PR-VEP 0.25° P100-N135 (μV)	13.10 (9.86, 14.88)	7.35 (3.59, 11.40)**	8.51 × 10^–4^

Data are expressed as the median (P25, P75).

Significant differences with Bonferroni correction of alcohol addiction group from the control group are indicated (**p < 9.09 × 10^–4^, ***p < 9.09 × 10^–5^, ****p < 9.09 × 10^–6^, Wilcoxon rank sum test).

### Significant visual pathway dysfunction was observed in alcoholic patients, even in those without clinically detectable retinopathy

Lastly, to verify whether retinal and optic nerve dysfunction happens earlier than clinically detectable retinopathy, we scanned their fundus photos. Among the 14 healthy control individuals, 8 have color fundus photos, and all (16 fundi) were normal. 4 of the 11 alcohol addicts had color photos of the fundus, and most of them (6 fundi) were generally normal, comparable to those of control (Supplementary Figure 1). In conclusion, functional alterations in visual pathway was observed in early stages of alcoholic vision impairment, even in those without detectable retinopathy. Neural deficits in alcohol addiction is not limited to neurons within the brain but is also evident in neurons and visual cells residing in the retina.

## Discussion

The eye is a sensitive and reliable marker of many brain diseases, including alcohol addiction. Chronic alcoholism can lead to many ophthalmic effects, such as alcohol-induced optic neuropathy, age-related macular degeneration (AMD), retinal vascular disease, glaucoma, and cataract (Karimi et al., 2021). Study showed that young adults with limited weekly habits of alcohol consumption already exhibited signs of visual dysfunction compared to control subjects of the same age (Brasil et al., 2015). Research also indicated that there was an insult to corneal endothelium with chronic alcohol use (Sati et al., 2018). The retina is an anatomical extension of the brain, and any changes in brain functioning and structure could be reflected in the retina. However, in clinical studies, there is still no conclusion on whether alcohol addiction causes damage to the retinal structure. Some determined that there were no statistically significant differences between the participants in retinal nerve fiber layer (RNFL) thickness, macular thickness, choroidal thickness, or ganglion cell-inner plexiform layer (GCIPL) thickness examined by Optical Coherence Tomography (OCT) (Ozsoy and Alim, 2020). In contrast, some discovered that that alcohol consumption significantly reduced the RNFL and macular thickness (Liu et al., 2021).

Whether the retina and visual system are functionally affected by alcohol have been even rarely reported. In humans, the acute effects of alcohol on the visual system have recently been evaluated with PR-VEP and revealed a P100 peak time delay (Kim et al., 2016). In rats, chronic alcohol intake induced a decrease of amplitude in ERG b-wave (Sancho-Tello et al., 2008). However, studies on the damage of retinal function in chronic alcohol addiction in human are very limited. Moreover, some previous studies using ERG or VEP were performed decades ago, and their measurements were different from those that are currently recommended by the ISCEV standards. Our study suggested that the PR-VEP peak times of N75, P100, and N135 increased significantly in chronic alcoholism (Figure 2B and Table 3). We also found that the amplitudes of ffERG b-waves but not a-waves were significantly decreased in alcohol addicts, especially the OPs displayed a marked reduction (Figure 1C and Table 2). In ERG, a-wave originates in the outermost retinal cells and depends upon the integrity of the outer region of the visual cells (Ruedemann, 1961). The b-wave depends upon the bipolar and the visual cells (Ruedemann, 1961). Dark-adapted OPs can reflect the response of the amacrine cells residing at middle retinal layers and can be used to assess retinal microcirculation (Luu et al., 2010; McCulloch et al., 2015). Therefore, our results indicate that a broad range of retinal cells are likely affected by alcohol use, particularly amacrine cells, visual cells and bipolar cells. Together, chronic alcohol addiction causes a functional decline both in the optic nerve and the retina in human beings, reflecting the retinal dysfunction in the early stage of alcohol-induced mild vision retardation.

Visual evoked potential may be affected by the abnormality of ERG. PR-VEP is the bioelectricity in the cerebral cortex generated by visual stimulation. It is extracted from the electroencephalogram (EEG) recorded on the scalp through signal averaging technology, and reflects the transmission of visual information from the retina to the visual center of the cerebral cortex. Normal VEP depends on the functional integrity of the visual pathway at all levels, including the eyeball, retina, retinal ganglion cells, optic nerve, optic radiation, and visual cortex. Therefore, abnormal VEP can occur when any part of the visual pathway is disrupted, such as the retina or the optic nerve. ffERG uses corneal electrodes to record the combined response of the entire retina to flash stimuli in both dark-adapted and light-adapted environments, and reflects the electrical activity of all retinal layers from photoreceptors to amacrine cells. Therefore, abnormality of ERG can lead to abnormality of VEP. Our study found that alcohol addiction impaired the function of the rod system, and the function of the inner retina, which could lead to the abnormality of PR-VEP.

Interpreting bulk visual electrophysiological data based on standard deviation may be inaccurate. In addition, using univariate statistical analyses only could overlook the correlation between parameters. For example, univariate statistical analyses assume that all the ERG parameters tested are independent; however, many of the ERG components were highly correlated, such as the amplitudes of the ERG a- and b-waves. Therefore, we used multivariate non-parametric statistical methods to analyze and present such bulk data, and found some highly sensitive electrophysiological parameters to alcohol addiction. The changes we observed may be indicative of disease severity and a more common non-dysmorphic alcohol-related visual pathway impairment, with clinical values in early prevention of alcoholic vision decline. In the future, more visual electrophysiological tests can be utilized to measure potential changes in the visual pathway of patients with alcohol addiction. For example, multifocal ERG (mfERG) can reflect the macular function with more precision in retinal anatomical positions. Photopic negative response (PhNR) can reflect the function of retinal ganglion cells, etc. These new techniques hold promising for early, differential, and more sensitive diagnosis of the toxicity of alcohol.

## Data availability statement

The raw data supporting the conclusions of this article will be made available by the authors, without undue reservation.

## Ethics statement

The studies involving human participants were reviewed and approved by the Peking University Third Hospital ethics committee. Written informed consent for participation was not required for this study in accordance with the national legislation and the institutional requirements.

## Author contributions

HZ, XX, and JH conceived the study and drafted the manuscript. XX performed all the electrophysiological tests. KF diagnosed the patients. XX and MZ collected the data. HZ performed the statistical analysis. HZ and JW interpreted the data. All authors contributed to the article and approved the submitted version.

## Supplementary material

Supplementary Figure 1 Representative images of subjects’ fundi are shown. Most alcohol addicts’ fundi are comparable to normal controls.
