# Supplementary figures and images for: Comprehensive visual electrophysiological measurements discover crucial changes caused by alcohol addiction in humans: Clinical values in early prevention of alcoholic vision decline
Source: Front Neural Circuits. 2022 Aug 11;16:912883. doi: 10.3389/fncir.2022.912883 (PMC9403052; doi:10.3389/fncir.2022.912883)

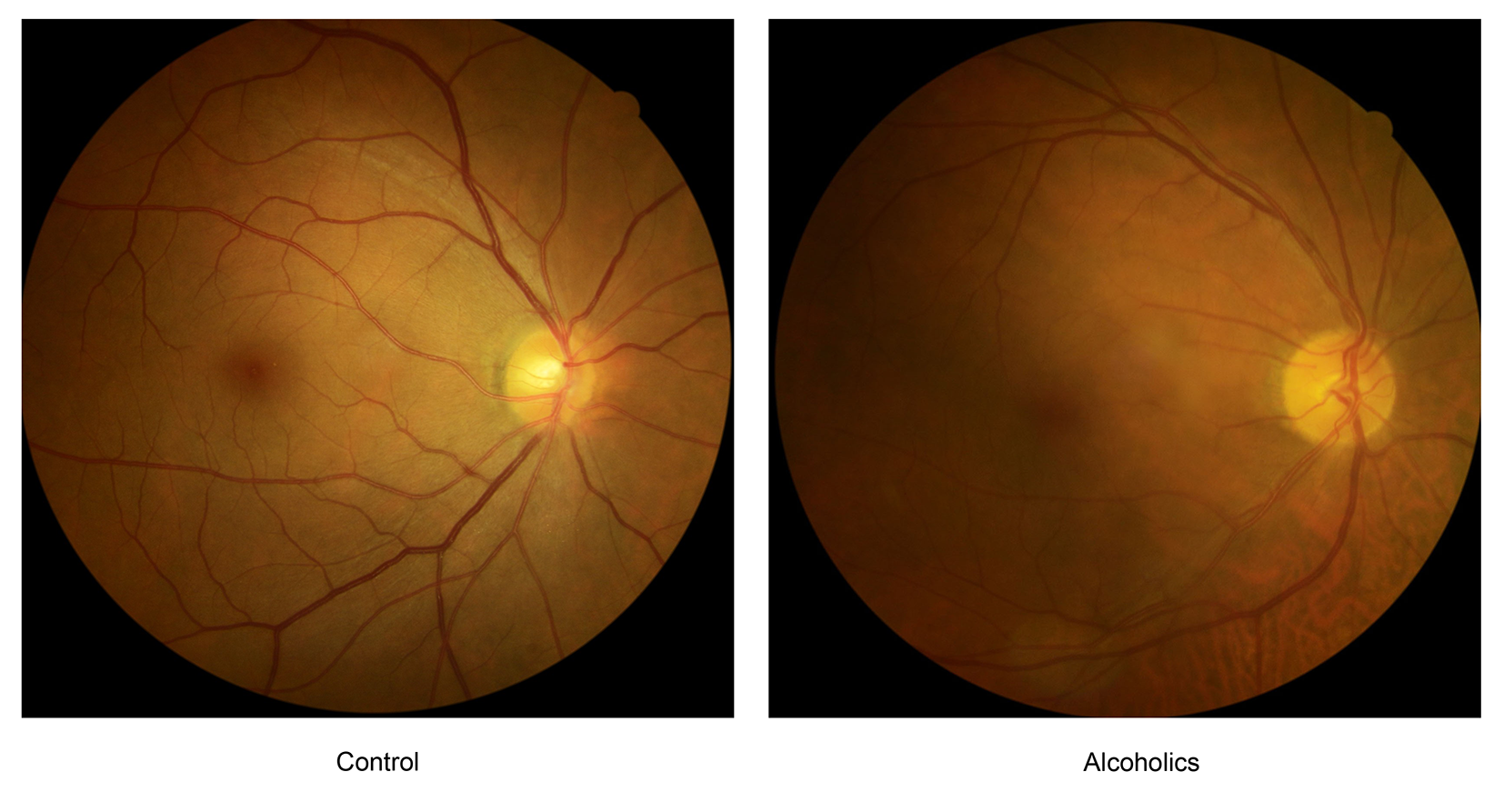

Supplement: Supplementary Figure 1 — Representative images of subjects’ fundi are shown. Most alcohol addicts’ fundi are comparable to normal controls. [file Image_1.TIF]
