# Supplementary material for: Comprehensive visual electrophysiological measurements discover crucial changes caused by alcohol addiction in humans: Clinical values in early prevention of alcoholic vision decline
Source: Front Neural Circuits. 2022 Aug 11;16:912883. doi: 10.3389/fncir.2022.912883 (PMC9403052; doi:10.3389/fncir.2022.912883)
Supplement: Supplementary file 2 [file Data_Sheet_1.docx]

**Supplemental Table 1a. Normality tests for ffERG**

Abbreviations: S, scotopic; P, photopic; OS: oscillatory; Flk: flicker.

| Characteristic | Normality tests for control | | Normality tests for alcoholics | |
| --- | --- | --- | --- | --- |
|  | W | *p*-value | W | *p*-value |
| S ffERG 0.01 b-wave (ms) | 0.91 | 0.26 | 0.87 | 0.09 |
| S ffERG 0.01 b-wave (μV) | 0.93 | 0.47 | 0.88 | 0.13 |
| S ffERG 3.0 a-wave (ms) | 0.76 | 4.28 × 10^-3^ | 0.63 | 1.31 × 10^-4^ |
| S ffERG 3.0 b-wave (ms) | 0.87 | 0.09 | 0.72 | 1.37 × 10^-3^ |
| S ffERG 3.0 a-wave (μV) | 0.77 | 7.16 × 10^-3^ | 0.73 | 2.26 × 10^-3^ |
| S ffERG 3.0 b-wave (μV) | 0.87 | 0.09 | 0.82 | 0.03 |
| S ffERG 3.0 b/a | 0.90 | 0.24 | 0.89 | 0.17 |
| S ffERG 10.0 a-wave (ms) | 0.80 | 0.02 | 0.75 | 3.58 × 10^-3^ |
| S ffERG 10.0 b-wave (ms) | 0.83 | 0.03 | 0.76 | 5.21 × 10^-3^ |
| S ffERG 10.0 a-wave (μV) | 0.85 | 0.06 | 0.77 | 5.86 × 10^-3^ |
| S ffERG 10.0 b-wave (μV) | 0.89 | 0.17 | 0.89 | 0.18 |
| S ffERG 3.0 OS P1 (ms) | 0.59 | 4.71 × 10^-5^ | 0.85 | 0.05 |
| S ffERG 3.0 OS N2 (ms) | 0.89 | 0.15 | 0.76 | 5.27 × 10^-3^ |
| S ffERG 3.0 OS P2 (ms) | 0.85 | 0.05 | 0.64 | 1.86 × 10^-4^ |
| S ffERG 3.0 OS N3 (ms) | 0.88 | 0.14 | 0.65 | 2.27 × 10^-4^ |
| S ffERG 3.0 OS P3 (ms) | 0.86 | 0.07 | 0.69 | 6.38 × 10^-4^ |
| S ffERG 3.0 OS N4 (ms) | 0.86 | 0.08 | 0.69 | 5.94 × 10^-4^ |
| S ffERG 3.0 OS P4 (ms) | 0.87 | 0.10 | 0.72 | 1.48 × 10^-3^ |
| S ffERG 3.0 OS1 (μV) | 0.94 | 0.50 | 0.80 | 0.02 |
| S ffERG 3.0 OS2 (μV) | 0.94 | 0.57 | 0.88 | 0.14 |
| S ffERG 3.0 OS3 (μV) | 0.91 | 0.29 | 0.92 | 0.37 |
| S ffERG 3.0 OS4 (μV) | 0.90 | 0.24 | 0.93 | 0.40 |
| S ffERG 3.0 OS Total (μV) | 0.85 | 0.06 | 0.87 | 0.09 |
| P ffERG 3.0 a-wave (ms) | 0.90 | 0.24 | 0.71 | 1.12 × 10^-3^ |
| P ffERG 3.0 b-wave (ms) | 0.85 | 0.05 | 0.77 | 6.38 × 10^-3^ |
| P ffERG 3.0 a-wave (μV) | 0.87 | 0.11 | 0.88 | 0.12 |
| P ffERG 3.0 b-wave (μV) | 0.86 | 0.09 | 0.72 | 1.45 × 10^-3^ |
| P ffERG 3.0 Flk P1 (ms) | 0.81 | 0.02 | 0.55 | 1.28 × 10^-5^ |
| P ffERG 3.0 Flk N1-P1 (μV) | 0.87 | 0.09 | 0.89 | 0.15 |
