# Supplementary material for: Comprehensive visual electrophysiological measurements discover crucial changes caused by alcohol addiction in humans: Clinical values in early prevention of alcoholic vision decline
Source: Front Neural Circuits. 2022 Aug 11;16:912883. doi: 10.3389/fncir.2022.912883 (PMC9403052; doi:10.3389/fncir.2022.912883)
Supplement: Supplementary file 3 [file Data_Sheet_2.docx]

**Supplemental Table 1b. Normality tests for PR-VEP**

| Characteristic | Normality tests for control | | | Normality tests for alcoholics | |
| --- | --- | --- | --- | --- | --- |
|  | W | *p*-value | W | | *p*-value |
| PR-VEP 1° N75 (ms) | 0.91 | 0.05 | 0.81 | | 7.32 × 10^-4^ |
| PR-VEP 1° P100 (ms) | 0.95 | 0.30 | 0.78 | | 2.51 × 10^-4^ |
| PR-VEP 1° N135 (ms) | 0.93 | 0.10 | 0.78 | | 2.71 × 10^-4^ |
| PR-VEP 1° N75-P100 (μV) | 0.83 | 1.38 × 10^-3^ | 0.94 | | 0.24 |
| PR-VEP 1° P100-N135 (μV) | 0.90 | 0.04 | 0.97 | | 0.61 |
| PR-VEP 0.25° N75 (ms) | 0.93 | 0.11 | 0.71 | | 3.03 × 10^-5^ |
| PR-VEP 0.25° P100 (ms) | 0.87 | 6.72 × 10^-3^ | 0.79 | | 3.42 × 10^-4^ |
| PR-VEP 0.25° N135 (ms) | 0.79 | 3.06 × 10^-4^ | 0.86 | | 4.79 × 10^-3^ |
| PR-VEP 0.25° N75-P100 (μV) | 0.91 | 0.05 | 0.94 | | 0.22 |
| PR-VEP 0.25° P100-N135 (μV) | 0.94 | 0.21 | 0.90 | | 0.03 |
