# Supplementary material for: Comprehensive visual electrophysiological measurements discover crucial changes caused by alcohol addiction in humans: Clinical values in early prevention of alcoholic vision decline
Source: Front Neural Circuits. 2022 Aug 11;16:912883. doi: 10.3389/fncir.2022.912883 (PMC9403052; doi:10.3389/fncir.2022.912883)
Supplement: Supplementary file 4 [file Data_Sheet_3.docx]

**Supplemental Table2a. Test of homogeneity of variance for ffERG**

| Characteristic | Levene's test | |
| --- | --- | --- |
|  | F | *p*-value |
| S ffERG 0.01 b-wave (ms) | 0.23 | 0.64 |
| S ffERG 0.01 b-wave (μV) | 1.36 | 0.26 |
| S ffERG 3.0 a-wave (ms) | 2.22 | 0.15 |
| S ffERG 3.0 b-wave (ms) | 1.00 | 0.33 |
| S ffERG 3.0 a-wave (μV) | 0.11 | 0.74 |
| S ffERG 3.0 b-wave (μV) | 1.10 × 10^-3^ | 0.97 |
| S ffERG 3.0 b/a | 3.36 | 0.08 |
| S ffERG 10.0 a-wave (ms) | 0.95 | 0.34 |
| S ffERG 10.0 b-wave (ms) | 0.23 | 0.64 |
| S ffERG 10.0 a-wave (μV) | 0.12 | 0.73 |
| S ffERG 10.0 b-wave (μV) | 0.06 | 0.81 |
| S ffERG 3.0 OS P1 (ms) | 3.13 | 0.09 |
| S ffERG 3.0 OS N2 (ms) | 1.00 | 0.33 |
| S ffERG 3.0 OS P2 (ms) | 2.47 | 0.13 |
| S ffERG 3.0 OS N3 (ms) | 1.73 | 0.20 |
| S ffERG 3.0 OS P3 (ms) | 1.24 | 0.28 |
| S ffERG 3.0 OS N4 (ms) | 0.53 | 0.48 |
| S ffERG 3.0 OS P4 (ms) | 0.69 | 0.42 |
| S ffERG 3.0 OS1 (μV) | 0.94 | 0.35 |
| S ffERG 3.0 OS2 (μV) | 1.63 | 0.22 |
| S ffERG 3.0 OS3 (μV) | 0.39 | 0.54 |
| S ffERG 3.0 OS4 (μV) | 0.61 | 0.45 |
| S ffERG 3.0 OS Total (μV) | 0.04 | 0.84 |
| P ffERG 3.0 a-wave (ms) | 4.10 | 0.06 |
| P ffERG 3.0 b-wave (ms) | 0.38 | 0.54 |
| P ffERG 3.0 a-wave (μV) | 0.14 | 0.71 |
| P ffERG 3.0 b-wave (μV) | 0.02 | 0.88 |
| P ffERG 3.0 Flk P1 (ms) | 0.30 | 0.59 |
| P ffERG 3.0 Flk N1-P1 (μV) | 1.18 | 0.30 |

Abbreviations: S, scotopic; P, photopic; OS: oscillatory; Flk: flicker.
