# Supplementary material for: Comprehensive visual electrophysiological measurements discover crucial changes caused by alcohol addiction in humans: Clinical values in early prevention of alcoholic vision decline
Source: Front Neural Circuits. 2022 Aug 11;16:912883. doi: 10.3389/fncir.2022.912883 (PMC9403052; doi:10.3389/fncir.2022.912883)
Supplement: Supplementary file 5 [file Data_Sheet_4.docx]

**Supplemental Table 2b. Test of homogeneity of variance for PR-VEP**

| Characteristic | Levene's test | |
| --- | --- | --- |
|  | F | *p*-value |
| PR-VEP 1° N75 (ms) | 8.90 | 4.74 × 10^-3^ |
| PR-VEP 1° P100 (ms) | 8.03 | 7.04 × 10^-3^ |
| PR-VEP 1° N135 (ms) | 4.11 | 0.05 |
| PR-VEP 1° N75-P100 (μV) | 1.70 | 0.20 |
| PR-VEP 1° P100-N135 (μV) | 0.03 | 0.86 |
| PR-VEP 0.25° N75 (ms) | 6.05 | 0.02 |
| PR-VEP 0.25° P100 (ms) | 9.68 | 3.35 × 10^-3^ |
| PR-VEP 0.25° N135 (ms) | 2.27 | 0.14 |
| PR-VEP 0.25° N75-P100 (μV) | 2.65 | 0.11 |
| PR-VEP 0.25° P100-N135 (μV) | 2.07 | 0.16 |
